# The Veterinary Profession: Its Relation to the Health and Wealth of the Nation

**Published:** 1897-09

**Authors:** 


### EDITORIAL.

### THE VETERINARY PROFESSION: ITS RELATION TO THE HEALTH AND WEALTH OF THE NATION.

The above is the comprehensive title of a pamphlet that has just been issued by the Veterinary Department of the University of Pennsylvania. This publication contains about twenty articles by distinguished alumni of the University of Pennsylvania, in which the various phases of veterinary work are discussed in all their bearings. It is illustrated by sixteen full-page, half-tone plates of scenes about the Veterinary Department and University.

The introductory article on "Veterinary Medicine" is by Dr. William Pepper, Professor of the Theory and Practice of Medicine and of Clinical Medicine. Dr. Pepper calls attention to the dignity and importance of veterinary work, its probable future, and the rewards that it offers for thorough students at the present time.

This pamphlet serves to show the great scope and value of veterinary science, and treats of a number of most important subjects that are too frequently lost sight of. The matters of diseases of animals transmissible to man, and meat-, milk-, and dairy-inspection as practised under national, State, and municipal direction are discussed in detail. The advantages of a veterinary training to the breeder and the physician are the subjects of two of the most interesting papers in the book. The development that may be expected in the science of horseshoeing, it is shown, must come from the veterinarian, and the advantages of such development are made manifest. The pleasures, trials, and rewards that fall to the lot of veterinarians practising in the East, South, and West, in city and country, are dwelt upon separately in articles that cannot fail to yield encouragement to the bulk of the profession, but to which no exception can possibly be taken because they are written by men who know whereof they speak; they are judicious and instructive. Veterinary science as it is taught in agricultural colleges, canine practice, a course in veterinary medicine preliminary to advanced work in medicine, the veterinary service in the United States Army, are subjects of short articles that are full of information and sound reasoning.

The entire book comprises eighty-eight pages. It is gotten up in the highest style of the printer's art—it is really a typographical gem—printed on the best of paper, the cover illuminated in the University colors—red and blue. The entire work cannot fail to make an excellent impression on everyone into whose hands it may come. It is safe to say that this is the most comprehensive, most reliable, and best publication on the subject so cleverly expressed by the title that has ever appeared in America. While it is issued by a veterinary school, it is in no sense a catalogue or prospectus, but a fund of information dealing with the veterinary profession in a new and original way and on a high plane.

This publication will do much to educate the public to a more complete recognition of the value of veterinary work, its general scope, usefulness, and possibilities, "its relation to the health and wealth of the nation."

